# SUMOylation of PPARγ by Rosiglitazone Prevents LPS-Induced NCoR Degradation Mediating Down Regulation of Chemokines Expression in Renal Proximal Tubular Cells

**DOI:** 10.1371/journal.pone.0079815

**Published:** 2013-11-08

**Authors:** Ying Lu, Qiao Zhou, Yongbing Shi, Jian Liu, Fang Zhong, Xu Hao, Cong Li, Nan Chen, Weiming Wang

**Affiliations:** 1 Department of Nephrology, Ruijin Hospital, Shanghai Jiao Tong University, Shanghai, China; 2 Department of Nephrology, Second Affiliated Hospital of Soochow University, Suzhou, China; University of Leuven, Rega Institute, Belgium

## Abstract

Rosiglitazone (RGL), a synthetic agonist for peroxisome proliferator activated receptor γ (PPARγ), exhibits a potent anti-inflammatory activity by attenuating local infiltration of neutrophils and monocytes in the renal interstitium. To evaluate the mechanisms that account for inhibiting inflammatory cells infiltration, we investigated the effect of RGL on chemokines secretion and nuclear factor-kappa B (NF-κB) activation in human renal proximal tubular cells (PTCs). We demonstrated that RGL significantly inhibited lipopolysaccharide (LPS)-induced interleukin-8 (IL-8) and monocyte chemoattractant protein-1 (MCP-1) production in a dose-dependent manner, without appreciable cytotoxicity. Chromatin immunoprecipitation (ChIP) assays clearly revealed that, RGL inhibited p65 binding to IL-8/MCP-1 gene promoters in LPS-stimulated PTCs. Interestingly, further experiments showed RGL reversed LPS-induced nuclear receptor corepressor (NCoR) degradation. In addition, knockdown of protein inhibitor of activated STAT1 (PIAS1), an indispensable small ubiquitin-like modiﬁer (SUMO) ligase, abrogated the effects of RGL on antagonizing LPS-induced IL-8/MCP-1 overexpression and NCoR degradation. These findings suggest that, RGL activates PPARγ SUMOylation, inhibiting NCoR degradation and NF-κB activation in LPS-stimulated PTCs, which in turn decrease chemokines expression. The results unveil a new mechanism triggered by RGL for prevention of tubular inflammatory injury.

## Introduction

Inflammatory cell infiltration in the renal interstitium is a prominent feature of a variety of progressive renal diseases. Infiltrating leukocytes produce proinflammatory and profibrotic cytokines that contribute to fibroblast proliferation, myofibroblast transdifferentiation, matrix production, and tubular atrophy. Recruiting leukocytes into the kidney involves local expression of chemotactic cytokines, that is, chemokines. Both vitro and vivo studies have demonstrated that proximal tubular epithelial cells are an important source of different cytokines/chemokines and thereby play a central role in regulation of the local inflammatory response[1,2]. Over-produced IL-8 (also known as CXCL8) and MCP-1 (also known as CCL2) mainly by proximal tubular epithelial cells initiate local infiltration of leukocytes, which are closely related to tubulointerstitial inflammation and fibrosis[2,3]. Therefore, unveiling the mechanisms modulating tubular epithelial cells secreting IL-8/MCP-1 may thus provide insights into development of therapeutic targets for renal disease. 

PPARγ is a ligand-activated nuclear receptor that has recently aroused interest because of its anti-inflammatory properties[4,5]. Rosiglitazone is one of the synthetic agonists for PPARγ. In vitro studies, treated with PPARγ agonist, has a potent anti-inflammatory effect by attenuating proinflammatory mediators expression, such as tumor necrosis factor α (TNFα), interleukin-1β (IL-1β), interleukin-12 (IL-12), matrix metalloproteinase-9 (MMP-9), and MCP-1[6,7]. In vivo studies, treated with PPARγ agonist, ameliorates diabetic and non-diabetic kidney disease by suppressing the recruitment of inflammatory cells into the renal interstitium[7-10]. However, very little is known about the effect of PPARγ agonist on production of IL-8 and MCP-1 by proximal tubular epithelial cells. 

Several mechanisms have been proposed to explain the anti-inflammatory properties of PPARγ agonist. First, PPARγ agonist interrupts NF-κB-dependent gene expression through covalent modifications of critical cysteine residues in inhibitor of κB (IκB) kinase (IKK) with subsequent prevention of IκB degradation and nuclear entry of NF-κB. The second mechanism by which PPARγ agonist interferes with NF-κB activation, direct inhibition of binding of NF-κB to target DNA without blocking IκB degradation and nuclear translocation of NF-κB. This is more likely to be achieved by alkylation of a conserved cysteine residue located in the DNA binding domain of Rel proteins (e.g. C38 in p65 and C62 in p50). Recent studies indicate that NCoR is also required for basal repression of a subset of NF-κB and activating protein-1 (AP-1) target genes, with loss of NCoR resulting in a partially activated phenotype in macrophages[11]. We note that a number of inflammatory response genes that are de-repressed in NCoR-deficient macrophages are also subject to transrepression by PPARγ agonists, suggesting a possible role of NCoR in this process. Recent studies of the mechanism by which PPARγ represses activation of the iNOS gene by LPS led to identification of a SUMOylation-dependent transrepression pathway[12]. SUMOylation of PPARγ by PIAS1, a indispensable small ubiquitin-like modiﬁer (SUMO) ligase, prevents NCoR degradation, thereby attenuating LPS-induced gene expression[12,13].

In the present study, we reported the effect of RGL on the expression of MCP-1 and IL-8 in LPS-stimulated renal tubular epithelial cells and identified a molecular pathway by which RGL represses transcriptional activation of inflammatory response genes , in order to develop a new pharmacological and therapeutic approache to prevention of tubular inflammatory injury.

## Materials and Methods

### Cell Culture

HK-2 cells, a primary human proximal tubular cell line, were purchased from the American Type Culture Collection (Rockville, MD) and were maintained in Gibco Keratinocyte-Serum-Free Medium supplemented with 5 ng/ml recombinant EGF and 0.05 mg/ml bovine pituitary extract (Invitrogen, Carlsbad, CA). These cells are not passaged forward beyond about 25-30 passages and were routinely cultured at 37 °C in a humidified atmosphere of 95% air-5% CO_2_ and nourished at intervals of 3-4 days. Cells were treated with 1 μg/ml LPS (from *Escherichia coli*) (Sigma, St Louis, Mo) with or without 10 µM RGL (Alexis, USA). In studies investigating the impact of GW9662 (Sigma, St Louis, Mo), an irreversible PPARγ antagonist, cells were treated with LPS (1 μg/ml) + RGL(10 µM)+GW9662 (10–100 µM).

### Cell viability assay

Methyl thiazolyl tetrazolium (MTT) was used as an indicator of cell viability as determined by the mitochondrial-dependent reduction to formazan. In brief, the cells were seeded and then treated with various reagents for the indicated time periods. After various treatments, the medium was removed and the cells were incubated with a solution of 0.5 mg/mL MTT (Sigma, St Louis, Mo). After incubation for 3 hours at 37 °C and 5% CO_2_, the supernatant was removed and the formation of formazan was observed by monitoring the signal at 540 nm using a microplate reader.

### RT-PCR and real-time RT-PCR

IL-8, MCP-1, NCoR, PIAS1 and glyceraldehyde 3-phosphate dehydrogenase (GAPDH) mRNA levels were determined by quantitative real-time RT-PCR. Total RNA was prepared from HK-2 cells by using TRIzol reagent (Invitrogen, Carlsbad, CA) according to the manufacturer protocol. Reverse transcription was performed using the standard reagent (Promega, Madison, MI) according to the manufacturer protocol. Real-time PCR amplification was performed using the SYBR Green master mix (Toyobo, Japan) and the Opticon 3 Real-time PCR Detection System (Bio-Rad, Hercules, CA). Primers of MCP-1, IL-8, NCoR, PIAS1 and GAPDH were as follows: IL-8 sense primer 5'-GAATTGAATGGGTTTGCTAGA-3', antisense primer 5'-CACTGTGAGGTAAGATGGTGG-3'; MCP-1 sense primer 5'-CAGCCAGATGCAATCAATGC-3', antisense primer 5'-GTGGTCCATGGAATCCTGAA-3'; NCoR sense primer 5'-GCTGATGAGGATGTGGATGG-3', antisense primer 5'-TTGGACTCTTGGATGTGCC-3'; PIAS1 sense primer 5'-TGCTAAAGGCTGGCTGTAGTC-3', antisense primer 5'-AGGGAGTAATGGCGATGATG-3'; GAPDH sense primer 5'-CAGGGCTGCTTTTAACTCTGGTAA-3', antisense primer 5'-GGGTGGAATCATATTGG A ACATGT-3'. Real-time RT-PCR was performed for 10 minutes at 95 °C followed by 44 cycles (denaturation for 15 seconds at 95°C, annealing with extension for 30 seconds at 60°C). Relative amounts of mRNA were normalized by GAPDH.

### Enzyme-linked immunosorbent assay

Cellular supernatants from all experimental conditions were collected, centrifuged to remove cell debris, and stored at -80°C for analysis. IL-8 and MCP-1 protein concentration were measured by commercial ELISA kits (Invitrogen, Carlsbad, CA) according to the manufacture protocol. Results were corrected for cell numbers.

### Western Blot Analysis

HK-2 cells were seeded onto 10 cm dish. After reaching confluence, cells were incubated with test substances, and then washed with cold sodium phosphate (PBS). For detection of NF-κB p50, p65 and NCoR, nuclear protein was extracted by NE-PER™ Nuclear and Cytoplasmic Extraction Reagents (Pierce, Rockford, IL) according to the manufacture protocol. Proteins (20 μg/lane) were separated by 10% sodium dodecyl sulfate polyacrylamide gels and electrotransferred to polyvinylidene difluoride (PVDF) membranes (Millipore, Bedford, MA). The membranes were blocked with TTBS buffer (50 mmol/L Tris HCl, 150 mmol/L NaCl, and 0.05% Tween 20; pH 7.5) containing 5% skim milk for 2 hours at room temperature, followed by incubating overnight at 4°C with a rabbit polyclonal anti-NFκB p50 antibody(1:1000 dilution; Santa Cruz, Inc., CA), a rabbit polyclonal anti-NFκB p65 antibody (1:1000 dilution; Santa Cruz, Inc., CA) or a rabbit polyclonal anti-NCoR antibody (1:1000 dilution; Santa Cruz, Inc., CA). After washing, a goat anti-rabbit IgG-horseradish peroxidase (HRP) (1:5000 dilution; KPL, USA) was added and incubated for 60 minutes at room temperature. Protein bands were visualized by enhanced chemiluminescence method (Millipore, Bedford, MA), and the band intensity was quantified by densitometry. To ensure equal loading, protein levels were normalized to the levels of LaminB detected using anti-Lamin B polyclonal antibody (1:2000 dilution; Santa Cruz, Inc., CA).

### Immunofluorescence staining

To test the localization of NF-κB, monolayer cells were cultured on glass coverslips in K-SFM. After pretreated with RGL for 2 hours, cells were stimulated with LPS (1 μg/ml) for 30 minutes, then fixed with 4% paraformaldehyde in PBS (pH 7.5) and permeabilized with 0.2% TritonX-100 for 5 minutes. The coverslips were incubated with polyclonal anti-p65 antibody (1:70 dilution; Santa Cruz, Inc., CA) over night at 4°C. They were then washed with PBS (PH7.5) and incubated with rhodamine-conjugated goat anti-rabbit IgG (1:2000 dilution; KPL, USA) for 2 hours at room temperature. After washing, coverslips with the stained cells were mounted in 80% glycerol in PBS, then photographed with a Nikon fluorescence photomicroscope.

### Preparation of Nuclear Protein Extracts and Electrophoretic Mobility Shift Assay (EMSA)

Nuclear protein was extracted by NE-PER™ Nuclear and Cytoplasmic Extraction Reagents (Pierce, Rockford, IL). EMSAs of nuclear protein extracts were performed as LightShift Chemiluminescent EMSA Kit (Pierce, Rockford, IL) given, using an biotin-labeled oligonucleotideprobe (Invitrogen, Carlsbad, CA). In control experiments, a mutate biotin-labeled oligonucleotide was added to the binding mixture before the addition of nuclear protein extract. 

### Chromatin Immunoprecipitation Assay (ChIP)

To confirm that the LPS-mediated NF-κB activation responsible for the higher IL-8 and MCP-1 expression, ChIP assays were performed using the EZ-ChIP kit (Millipore, Bedford, MA), according to manufacturer’s instructions with slight modifications. After treatment of the cells, 37% fresh formaldehyde was added directly into the medium at a final concentration of 1% formaldehyde and incubated for 10 minutes at room temperature, followed by quenching with 125 mM glycine. The cells were then scraped using 2 ml prechilled PBS containing 1 × protease inhibit. The cell pellet was harvested by spinning at 700 g at 4°C, and lysis buffer was added (provided in the kit) to harvest nuclei. DNA was then sheared by sonication. A total of 100 μ l of the sheared crosslinked chromatin was then mixed with 60 μ l protein G agarose beads and 10 μg of immunopreciptating antibody against p65 (Santa Cruz, Inc., CA), or normal mouse IgG (as a negative control) diluted in 450 ml dilution buffer overnight at 4°C. The agarose beads binding antibody-chromatin complex was then washed with 0.5 ml each of a series of cold wash buffers in the order of low salt buffer, high salt buffer, LiCl buffer, and Tris-EDTA buffer. The crosslinking of protein-DNA complexes were reversed to free DNA by incubation at 65°C for 4 hours and purified using DNA purification spin columns following the manufacturer ’s instructions. Finally, DNA samples were then amplified by PCR using the following primers against the NF-kB site in IL-8 promoter: 5'-AATCGTGGAATTTCCTCTGACA-3' (sense) and 5'-GTTTCTTCCTGGCTCTTGTCCT-3' (antisense), MCP-1 promoter: 5'-TATGCCTTTGTCCAAGTC TGA-3' (sense) and 5'-GGAAGCGAGGAAACTAGATGA-3' (antisense). The PCR conditions were denaturation at 94°C for 3 minutes and 32 cycles of denaturation at 94°C for 15 seconds, annealing at 55°C for 20 seconds, and extension at 72°C for 20 seconds. An additional extension step of 2 minutes at 72°C was carried out after the last cycle. PCR products were subjected to electrophoresis in a 4% agarose gel in TBE buffer and analyzed densitometrically.

### Transient Transfection

HK-2 cells were seeded onto six-well plates containing 2 ml of growth medium for 24 hours. siRNA was transfected into HK-2 cells using Lipofectamine^TM^2000 (Invitrogen, Carlsbad, CA) according to the manufacturer protocol. Briefly, diluting 100pmol scrambled RNA (sicontrol) or siRNA for NCoR or PIAS1 in 250 µl Opti-MEM (Invitrogen, Carlsbad, CA) (final concentration of RNA when added to the cells is 30 nM). Then diluting 5 µl Lipofectamine™2000 in 250 µl Opti-MEM, incubating for 5 minutes at room temperature. After the 5 minutes incubation, combine the diluted oligomer with the diluted Lipofectamine™2000. After the incubation for 20 minutes, the oligomer-Lipofectamine™ 2000 complexes were added to each well replacing the growth medium. 

### Statistical analysis

All the results were expressed as means ± SD of three assays. One-way ANOVA multiple comparison tests were applied to evaluate the statistical differences between the results. *P* < 0.05 was considered statistically significance.

## Results

### Assessment of cell toxicity of rosiglitazone

**Figure 1:**
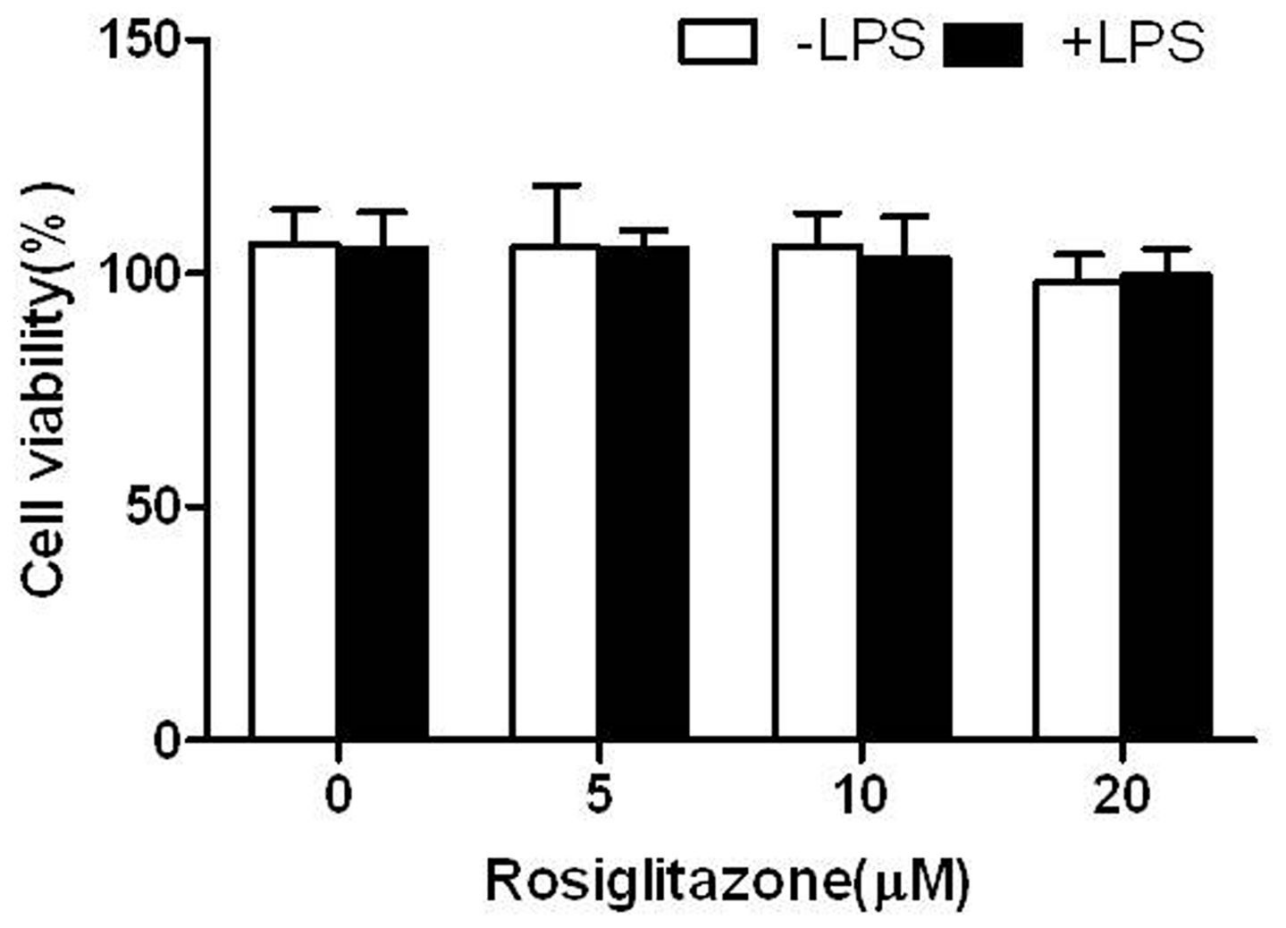
Cytotoxic assessment of RGL in HK-2 cells. HK-2 cells were treated with the indicated concentrations of RGL (0, 5, 10 , 20 μM) for 24 hours with or without 1 μg/ml LPS. HK-2 cells viability was assessed using an MTT assay, and the surviving cell values are expressed as a percent of control treated cells (no addition of RGL). Each value indicates the mean ± SD from three independent experiments.

To exclude the possibility that reductions of the levels of inflammatory cytokine from the cells was due to direct toxicity of RGL to the cells, we evaluated cell toxicity of RGL at different concentrations (0-20 μM) for 24 hours using MTT assay. RGL-induced cell toxicity was negligible at concentrations of 0-20 μM in HK-2 cells (Figure 1).

### Rosiglitazone inhibits LPS-induced IL-8 and MCP-1 production via a PPARγ-dependent mechanism in HK-2 cells

**Figure 2:**
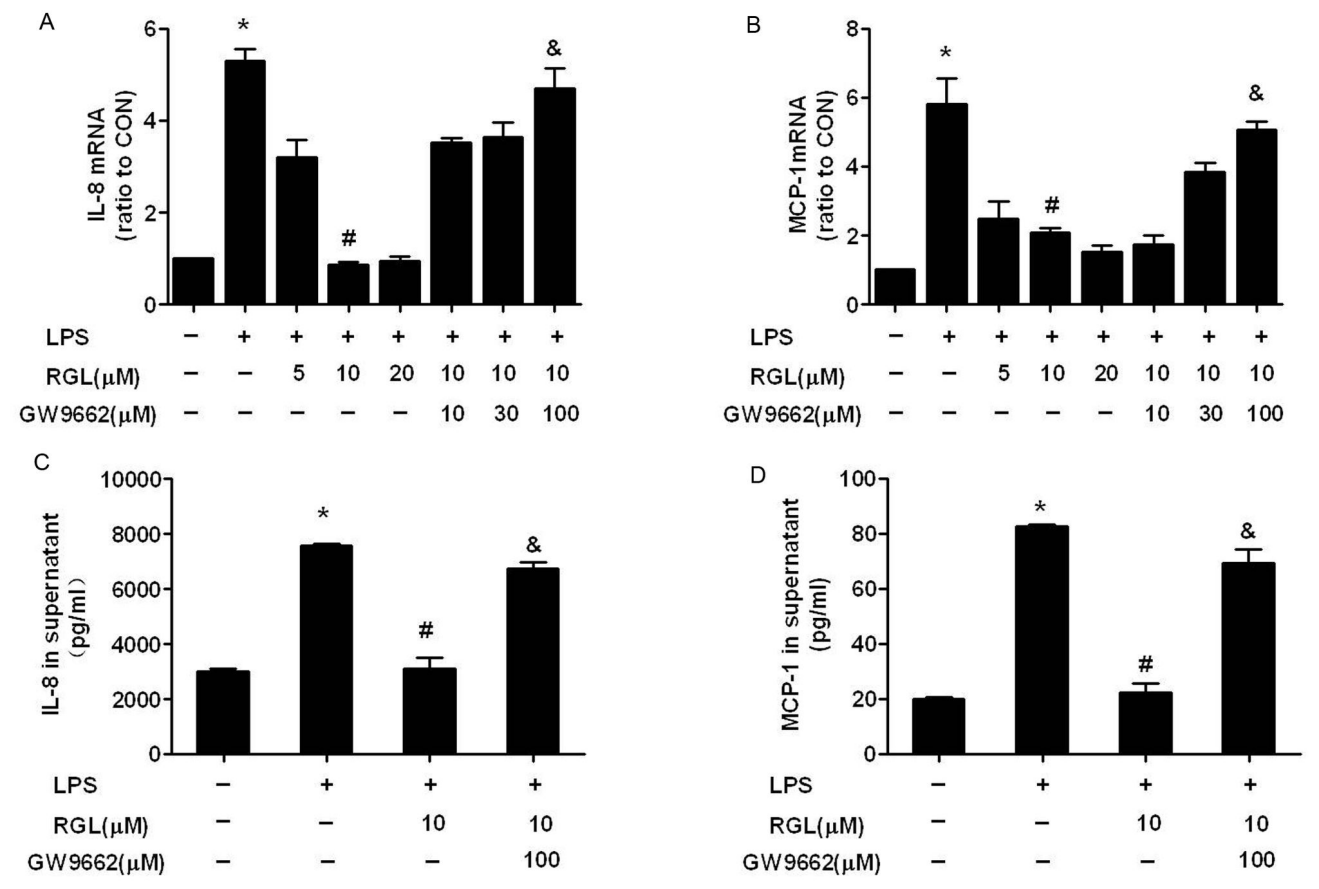
Rosiglitazone inhibits IL-8 and MCP-1 production via a PPARγ-dependent mechanism in LPS-stimulated HK-2 cells. (A, B) HK-2 cells were pretreated with rosiglitazone (5, 10, 20 µM) in the absence or presence of GW9662 (10, 30, 100 µM) for 2 hours and then treated with 1 μg/ml LPS for 4 hours. IL-8 and MCP-1 mRNA was analyzed by real-time PCR. (C, D) HK-2 cell were pretreated with 10 µM rosiglitazone in the absence or presence of 100 µM GW9662 for 2 hours and then treated with 1 μg/ml LPS for 24 hours. IL-8 and MCP-1 protein in cell supernatants was measured by ELISA. Results are shown as mean ± SD and representative of three independent experiments. *P<0.05 compared to the control group; ^*#*^ *P*<0.05 compared to the LPS-treated group; ^*&*^ *P*<0.05 compared to LPS + RGL (10 µM)-treated group.

To assess the effect of LPS on IL-8 and MCP-1 production, HK-2 cells were stimulated with LPS (1 μg/ml). The results showed that, in response to LPS for 4 hours, IL-8 and MCP-1 mRNA increased to 5.30±0.45 and 5.80±1.29-fold (Figure 2A, B) compared to control cells (no addition of LPS, RGL and GW9662). Accordingly, after application of LPS for 24 hours, IL-8 and MCP-1 protein in cell supernatants increased to 2.39±0.18 (Figure 2C) and 3.11±0.47-fold (Figure 2D), respectively. To evaluate the influence of RGL on LPS-induced IL-8 and MCP-1 production, HK-2 cells were treated with RGL, in the absence or presence of GW9662, an irreversible PPARγ antagonist. Figure 2A and 2B shown, RGL (concentration increased from 5 to 20 µM) significantly inhibited LPS-induced IL-8 and MCP-1 mRNA expression. RGL (10 µM) also exerted similar inhibitory effects on LPS-induced IL-8 and MCP-1 protein release into supernatant (Figure 2C, D). GW9662 (100 µM) rebounded the mRNA and protein level of MCP-1 and IL-8 in presence of RGL, indicating RGL inhibited IL-8 and MCP-1 expression via a PPARγ-dependent manner.

### Rosiglitazone fails to reverse LPS-induced NF-κB nuclear translocation, but inhibits NF-κB binding in IL-8/MCP-1 promoters

**Figure 3:**
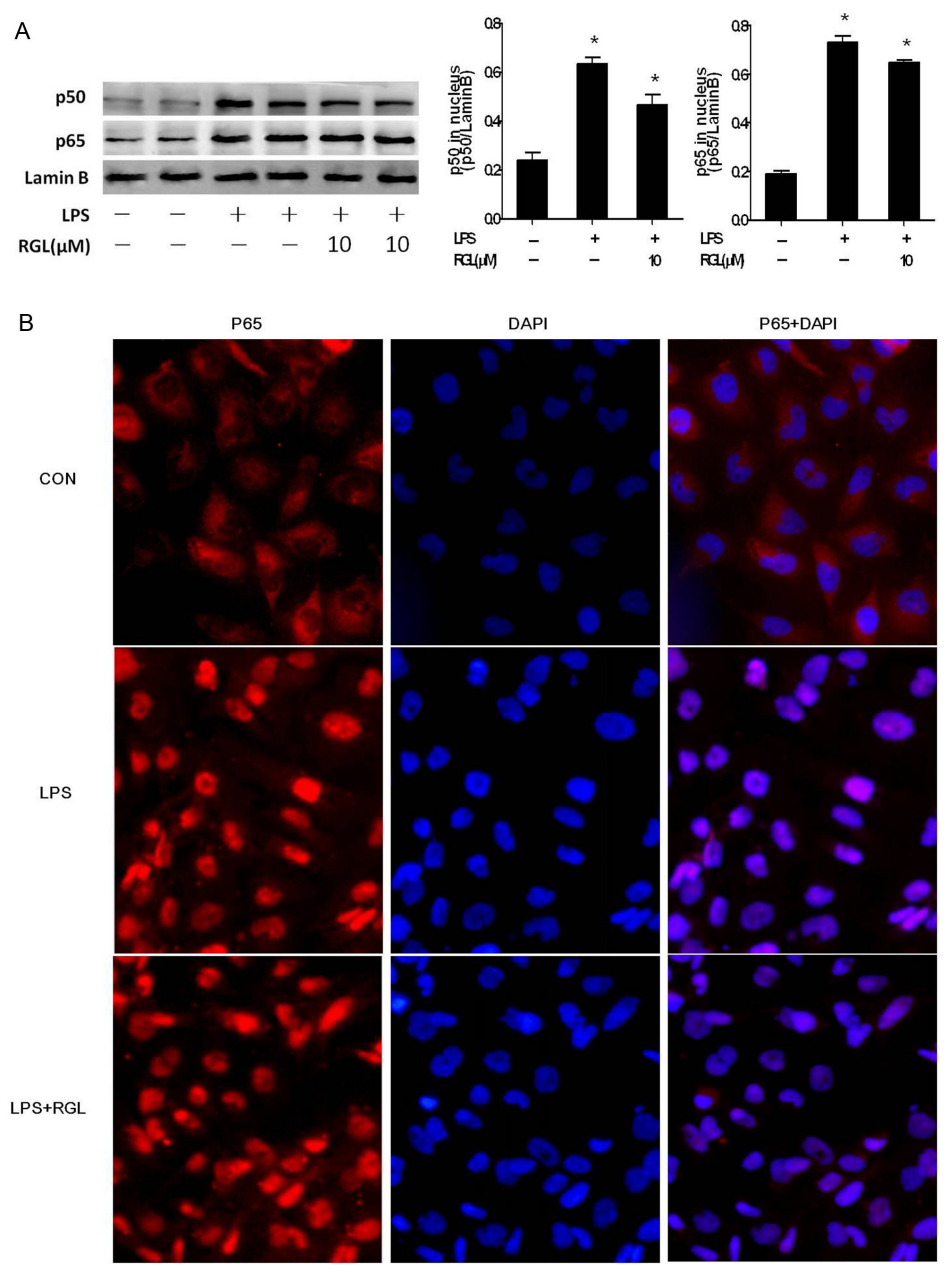
Rosiglitazone fails to reverse LPS-induced NF-κB nuclear translocation. HK-2 cells were treated without or with 10 µM RGL for 2 hours and then stimulation with 1 μg/ml LPS for 30 minutes. Nuclear protein was extracted for immunoblotting or cells was processed for immunofluorescent staining as described in 'Material and Methods'. (A) Relative levels of p50 and p65 in nucleus are determined by densitometric analysis and are presented as the relative ratio of LaminB, respectively. The 2 lanes for the same group in the blot represent duplicate experiments. The column bar graph shows the means ±SD of values obtained by densitometric analysis of three independent experiments. *P<0.05 compared to the control group. (B) Immunofluorescent staining shows that NF-κB p65 translocated into nucleus after treatment of 1 μg/ml LPS for 30 minutes and pretreatment of 10 µM RGL fialed to antagonize p65 nuclear translocation (Original magnification × 400).

NF-κB plays a central role in the regulation of cytokines and chemokines expression. Initially, the objective was to determine the status of NF-κB in LPS-stimulated HK-2 cells. For this, we examined NF-κB nuclear translocation by using Western-blot analysis of nuclear extracts. As shown in Figure 3A, treatment of LPS (1 μg/ml) for 30 minutes markedly increased the levels of p50 and p65 subunit in nuclear extracts when compared to unstimulated cells. RGL (10 µM) failed to inhibit p50 and p65 translocation from cytosol into nucleus (Figure 3A). To address the translocation of NF-κB, NF-κB was detected by immunofluorescent staining. As shown in Figure 3B, treatment of LPS (1 μg/ml) significantly increased p65 translocation into nucleus, and RGL failed to attenuate LPS-induced p65 nuclear translocation. 

**Figure 4:**
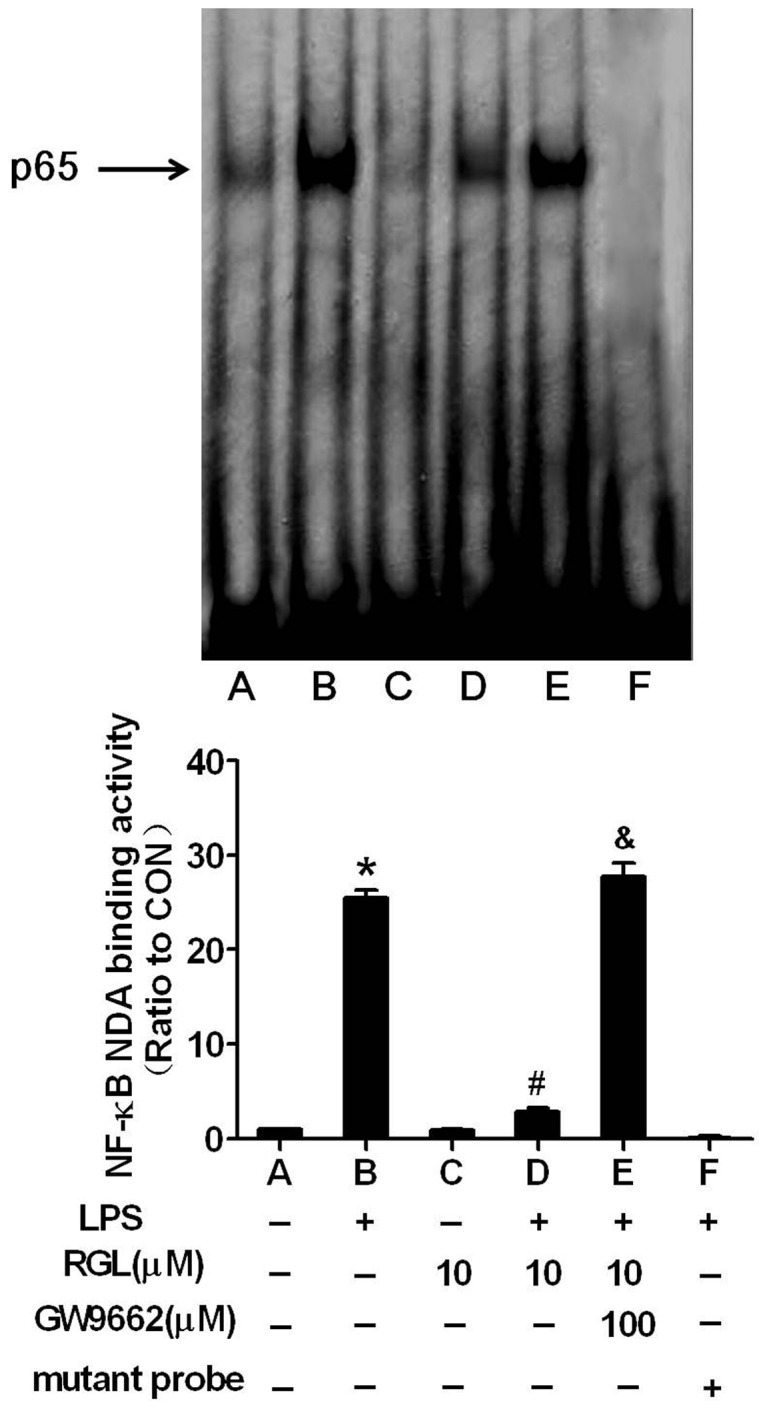
Rosiglitazone inhibits NF-κB-DNA binding activity. (A) HK-2 cells were pretreated with 10 µM rosiglitazone in the absence or presence of 100 µM GW9662 for 2 hours and then treated with 1 μg/ml LPS for 30 minutes. Equal amounts of nuclear extracts were assayed for binding a biotin-labeled double-stranded NF-κB oligonucleotide as described in 'Material and Methods'. The positions of NF-κB p65 was indicated. The column bar graph shows the means ± SD of values obtained by EMSAs densitometric analysis from three independent experiments. *P<0.05 compared to the control group; ^*#*^ *P*<0.05 compared to the LPS-treated group; ^*&*^ *P*<0.05 compared to LPS + RGL (10 µM)-treated group.

Next, we performed electrophoretic mobility shift assay to investigate NF-κB DNA-binding activity. Figure 4 shown that incubation of HK-2 cells with LPS-containing medium increased NF-κB DNA-binding activity. The increase in DNA-binding activity of NF-κB was completely suppressed by RGL. GW9662 (100 µM) drained the effect of RGL, indicating PPARγ is indispensable for RGL antagonizing LPS-induced NF-κB activation. 

**Figure 5:**
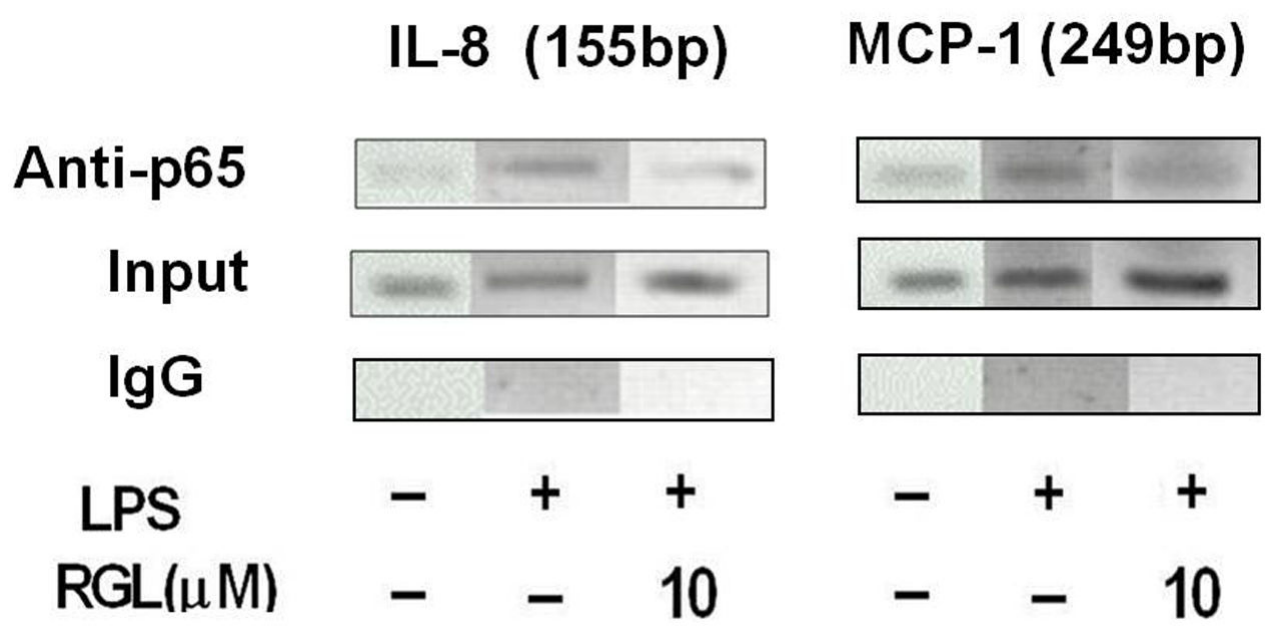
Rosiglitazone inhibits NF-κB binding in IL-8/MCP-1 promoters. HK-2 cells were treated without or with 10 µM RGL for 2 hours and then stimulation with 1 μg/ml LPS for 60 minutes. The representative agarose gel show PCR products of DNA obtained after chromatin immunoprecipitation with anti-p65 antibody (IP:anti-p65), non -immunoprecipitated DNA (input), or normal mouse IgG (negative control) amplified with primers specific for NF-κB site in IL-8/MCP-1 promoter.

To further obtain direct evidence that IL-8 and MCP-1 are the direct NF-κB target genes induced by LPS stimulation and RGL prevents the binding of NF-κB to IL-8/MCP-1 promoters, ChIP assays were performed using primers from NF-κB binding sites in IL-8/MCP-1 promoter. These experiments revealed LPS induced p65 binding in IL-8/MCP-1 promoter (Figure 5), along with preceding data, that substantiated the role of NF-κB in LPS-mediated upregulation of IL-8 and MCP-1. The increase in NF-κB binding to IL-8/MCP-1 promoter was completely suppressed by RGL, confirming activation of PPARγ downregulated LPS-induced NF-kB activation (Figure 5).

### Rosiglitazone inhibits LPS-induced IL-8 and MCP-1 expression in a NCoR-dependent manner

**Figure 6:**
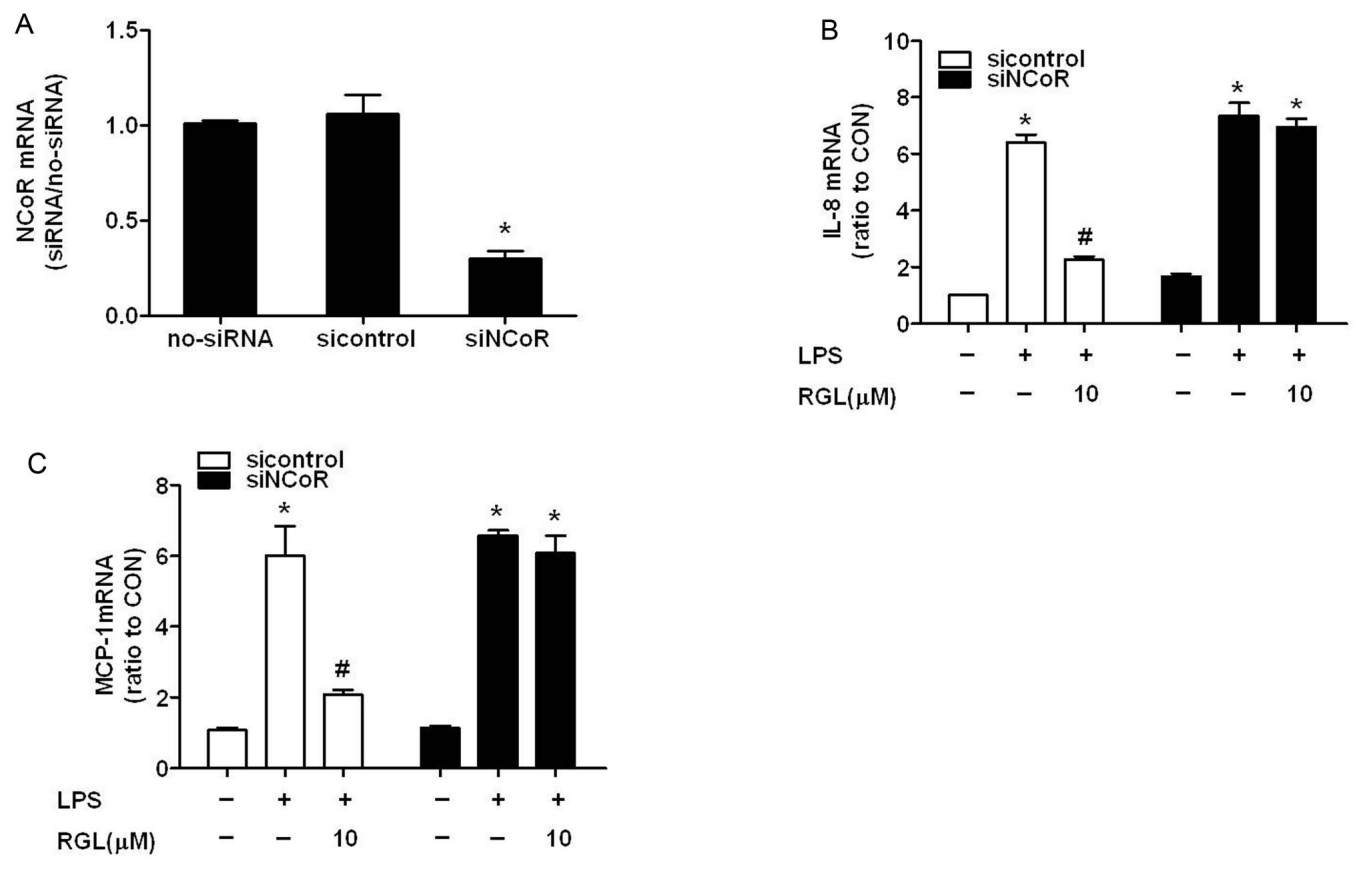
RGL inhibits LPS-induced IL-8 and MCP-1 expression in a NCoR-dependent manner. HK-2 cells were transfected with siNCoR to knock down NCoR, or transfected with sicontrol as controls. Two days after transfection, IL-8 and MCP-1 mRNA were quantified after incubated without or with 10 µM RGL for 2 hours followed by stimulation with 1 μg/ml LPS for 4 hours in HK-2 cells and HK-2/NCoR-knockdown cells. (A) Knockdown efficience of NCoR mRNA by siRNA. (B) The expression of IL-8 mRNA in different groups. (C) The expression of MCP-1 mRNA in different groups. The results are representative of three independent experiments. *P<0.05 compared to the control group; ^*#*^ *P*<0.05 compared to the LPS-treated group.

Recent studies indicate that a number of inflammatory response genes that are de-repressed in NCoR-deficient macrophages are subject to transrepression by PPARγ agonists suggesting a possible role of NCoR in this process. To better characterize the relation between beneficial effects of RGL above and NCoR, NCoR was knocked down by a specific siRNA. SiRNA-induced NCoR depletion (82.48% Figure 6A) significantly antagonize the inhibitory effects of RGL on LPS-induced IL-8 and MCP-1 mRNA expression(Figure 6B, C). The data above indicated that NCoR was indispensable for RGL suppressing chemokines overexpression.

### Rosiglitazone prevents LPS-induced NCoR degradation in HK-2 cells

**Figure 7:**
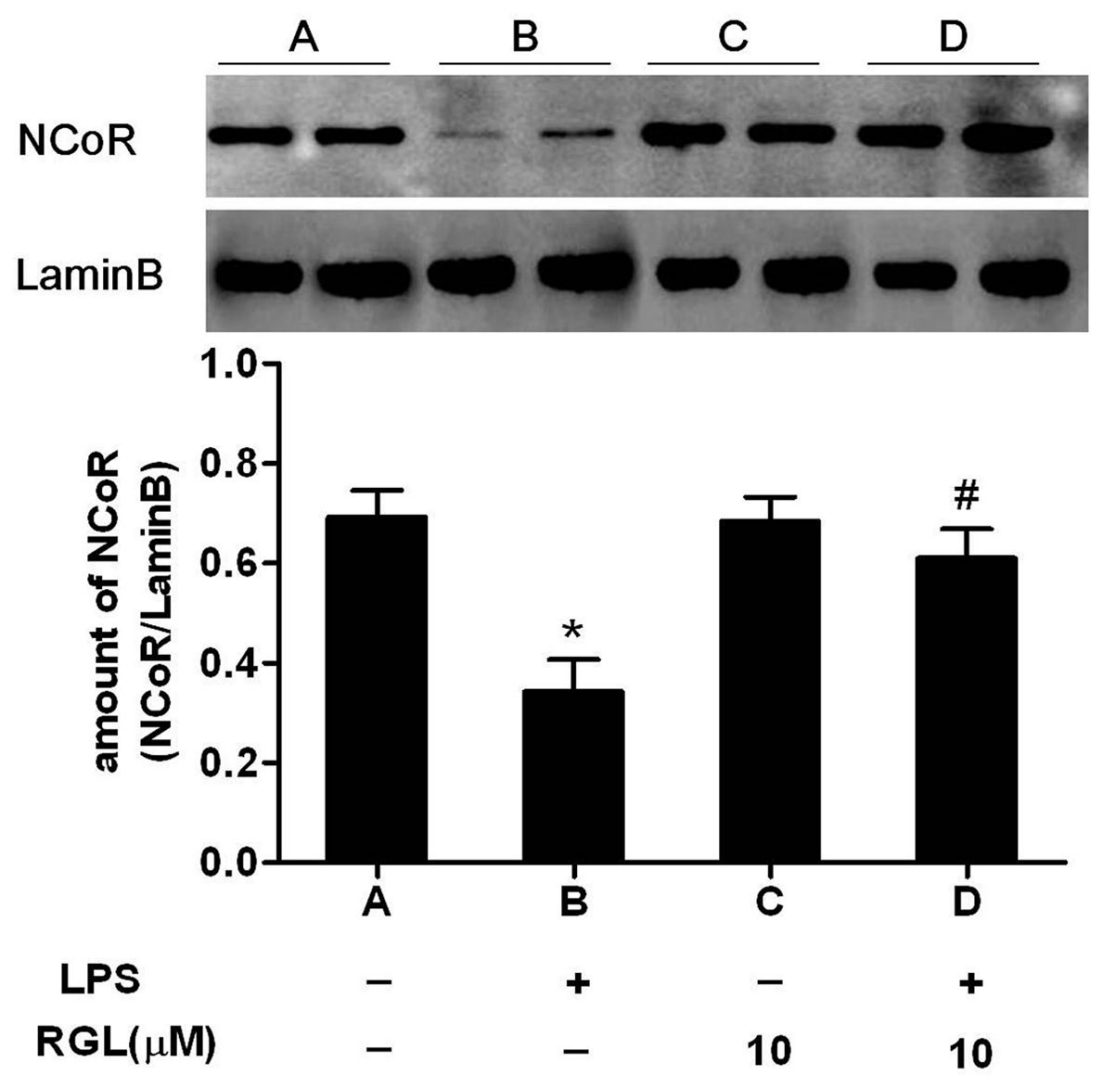
RGL abrogates LPS-induced NCoR degradation in HK-2 cells. HK-2 cells were treated without or with 10 µM RGL for 2 hours and then stimulation with 1 μg/ml LPS for 30 minutes. Nuclear protein was extracted and NCoR was quantitated by immunoblotting analysis in different groups. The column bar graph shows the means ± SD of values obtained by blot densitometric analysis from three independent experiments. *P<0.05 compared to the control group; ^*#*^ *P*<0.05 compared to the LPS-treated group.

Figure 7 shown, NCoR protein was significantly reduced in LPS group (reduced to 49.54% compared to control group). Incubating with RGL for 2 hours prior to LPS (1μg/mL), reversed LPS- induced NCoR degradation. 

### SUMOylation of PPARγ by rosiglitazone antagonizes LPS-induced NCoR degradation

**Figure 8:**
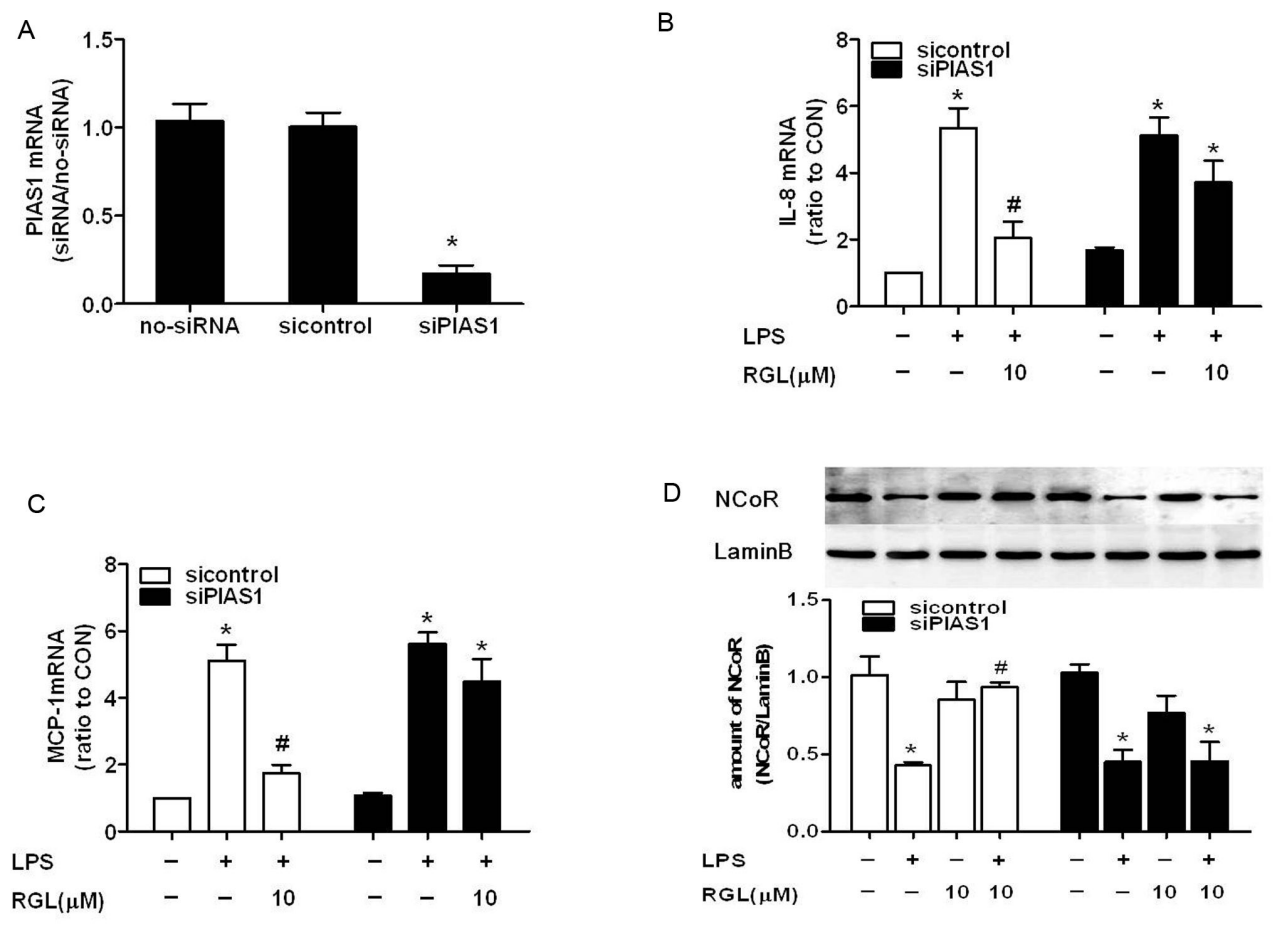
SUMOylation of PPARγ by RGL antagonizes LPS-induced chemokine expression and NCoR degradation. HK-2 cells were transfected with siPIAS1 to knock down PIAS1, or transfected with sicontrol as controls. Two days after transfection, IL-8 and MCP-1 mRNA were quantified after incubated with 10 µM RGL for 2 hours followed by stimulation with LPS 1 μg/ml for 4 hours in HK-2 cells and HK-2/PIAS1-knockdown cells. Quantification of mRNA level was performed by real-time PCR. (A) Knockdown efficience of PIAS1 mRNA by siRNA. (B) The expression of IL-8 mRNA in different groups. (C) The expression of MCP-1 mRNA in different groups. (D) Two days after transfection, Nuclear protein was extracted after incubated with 10 µM RGL for 2 hours followed by stimulation with 1 μg/ml LPS for 30 minutes in HK-2 cells. NCoR protein were quantitated by immunoblotting analysis in different groups. The results are representative of three independent experiments. *P<0.05 compared to the control group; ^*#*^ *P*<0.05 compared to the LPS-treated group.

PIAS1 belongs to the SUMO E3 ligase family and is an indispensable ligase for PPARγ SUMOylation. Pascual et al. show that SUMOylated PPARγ prevents NCoR degradation by the ubiquitination/19S proteasome machinery. To establish whether RGL inhibits NCoR degradation by SUMOylating PPARγ, we knocked down PIAS1 by a specific siRNA. 48 hours after transfection, HK-2 cells were exposed to RGL and LPS as described above. SiRNA-induced PIAS1 depletion (81.65%, Figure 8A) did not affect LPS-induced IL-8 and MCP-1 expression, but significantly abrogated RGL capable of attenuating IL-8 and MCP-1 expression(Figure 8B, C). Moreover, RGL failed to reverse NCoR degradation induced by LPS (Figure 8D) in PIAS1-deficient HK-2 cells. The results suggest that RGL-mediated PPARγ SUMOylation is responsible for suppressing chemokines overexpression and NCoR degradation.

## Discussion

In the present study, we provide evidence, for the first time, that RGL inhibits chemokines overexpression in HK-2 cells via a PPARγ-dependent way. The beneficial action of RGL is associated with PPARγ SUMOylation, thereby suppressing LPS-induced NCoR degradation, and subsequently inhibiting NF-κB DNA-binding activity in HK-2 cells. 

Kidney disease is usually associated with interstitial leukocytic cell infiltrates, which may contribute to disease progression by production of proinflammatory, proapoptotic, and profibrotic mediators. Inflammation in tubulointerstitum plays an important role and is closely associated with the prognosis of kidney diseases. Recent advances in the understanding of the molecular mechanisms that regulate renal inflammatory cell recruitment suggest chemokines and chemokine receptors as new targets for specific pharmacological intervention. It has been demonstrated that chemokines including MCP-1 and IL-8 are related to tubulointerstitial lesions[14,15]. In both experimental diabetic and nondiabetic chronic kidney disease these chemokines are overexpressed, which correlates with inflammatory cell infiltrates[14-16]. Recent studies have suggested that renal tubular epithelial cells are the key cells which secreted such chemokines. These findings suggest that intervention strategies to prevent renal tubular epithelial cells secreting chemokines facilitate a reduction in tubulointerstitial inflammation lesion. The present study shown, RGL, a synthetic PPARγ agonist, diminished LPS-induced IL-8 and MCP-1 overexpression via a PPARγ-dependent way in renal tubular epithelial cells, different from 15d-PGJ_2_, a endogenous agonist of PPARγ, exerting anti-inflammatory effect via a PPARγ-independent mechanism[17]. Our Study have consistented with previous reports that PPARγ agonists exert beneficial effects on renal injury through an anti-inflammatory mechanism [18,19]. But potential mechanisms whereby RGL ameliorating inflammatory gene expression is less clear. 

PPARγ is one of the members of the nuclear hormone receptor superfamily that is expressed in variety of cells, including renal tubular epithelial cells, mesangial cells, endothelial cells and podocytes. PPARγ, which function as ligand-dependent transcription factor, can heterodimerize with retinoid X receptors and then bind to PPAR-responsive elements (PPRE) in target gene promoters, usually leading to transcriptional activation[4]. Through this mechanism, PPARγ agonists can control a wide range of physiological processes. In addition to acting directly to stimulate PPARγ-dependent gene expression, and like glucocorticoid receptor agonists, PPARγ agonists have also been shown to negatively regulate infalmmatory cytokine gene expression[17,20]. Because cellular signaling pathways activated during inflammation almost always lead to activation of the transcription factor NF-κB, agents that modulate the activity of this transcriotion factor are of great interest as potential therapeutics, especially those with anti-inflammatory properties[20,21]. Although significant controversy exists in the literature and several distinct hypotheses have been proposed, the preponderance of evidence points to intranuclear crosstalk between PPARγ and NF-κB, including affecting “upstream” signaling intermediates and obstructing transcription in a phenomenon known as trans-repression[4,5]. Straus et al.[17] reported that 15d-PGJ_2_ could inhibit the expression of cytokines by blocking NF-κB translocation into nucleus via repressing IκBα phosphorylation. In the current study, we demonstrate that RGL fail to inhibit NF-κB nuclear translocation, but significantly inhibit NF-κB binding in IL-8/MCP-1 promoters, indicating that RGL inhibit chemokines expression via a trans-repression mechanism.

NCoR is one of a corepressor complex, containing transducin β-like protein-1 (TBL1), and histone deacetylase-3 (HDAC3), with the latter one mediating transcriptional repression[22]. In unstimulated cells, PPARγ interact with NCoR, which serves to actively repress PPARγ-mediated transcription[22]. After being stimulated with PPARγ ligands, the repressive NCoR dissociates from PPARγ and is replaced by coactivaor complexes containing proteins such as steroid receptor coactivator-1 (SRC1) and p300/CREB-binding protein (CBP), which lead to expression of gene regulated by PPARγ[22]. Further to investigate the effect of NCoR, we knocked down NCoR by siRNA. We assumed that silencing NCoR amplified the anti-inflammation action of RGL. Beyond our expectation, in NCoR-silenced HK-2 cells, RGL fail to attenuate LPS-induced chemokines overexpression. Previous study reports, that in the unstimulated state, NF-κB-regulated proinflammatory gene promoters are actively repressed by binding of NCoR/HDAC3[12]. Induction of NF-κB by agents such as LPS results in degradation of NCoR and promoter transcription[12]. The present study demonstrates that RGL significantly restore LPS-induced degradation of NCoR, indicating that the anti-inflammatory action of RGL is mediated by preventing NCoR degradation, and NCoR is indispensable to the anti-inflammatory action. 

The inhibitory action of nuclear receptors on inflammatory pathways has been best studied in the case of the glucocorticoid receptor (GR). Antagonism of the NF-κB and AP-1 signaling pathways by GR occurs by several distinct mechanisms, including direct interactions with p65 and c-Jun, competition for limited amounts of transcriptional coactivators, chromatin remodeling, and by induction of the NF-κB inhibitor, IκB[17,22]. In contrast to GR, the mechanism whereby retinoid X receptors (RXRs) heterodimeric ameliorated receptors such as PPAR inhibit inflammation is less clear. Pascual et al.[12] reports that PPARγ agonists, which induce SUMOylation of PPARγ, prevent LPS-induced NCoR degradation and lead to transrepresson of the NF-κB-dependent promoters, thereby maintaining the actively repressed state and effectively blocking cytokine expression. In our study, we knocked down PIAS1, which is indispensable to SUMOylation of PPARγ, to prevent PPARγ SUMOylation. In this study, silencing PIAS1 in HK-2 cells, abrogates the anti-inflammatory effects of RGL. In addition, RGL fails to reverse LPS-induced NCoR degradation in PIAS1-knockdown cells. The data above supports, that the previous assumption that PPARγ SUMOylation in response to RGL regulates the activity of NF-κB via preventing NCoR from degradation. 

In conclusion, the present study shows that the PPARγ agonist, RGL, decreased the secretion of chemokines in LPS–stimulated renal tubular epithelial cells by inhibiting NF-κB activation via a PPAR-dependent mechanism, which was associated with SUMOylation of PPARγ. Since the NF-κB system is an important regulator of the response of epithelial cells to injury, RGL may represent a key pharmacological target for ameliorating infammation-associated kidney disease.
